# Phage Interactions with the Nervous System in Health and Disease

**DOI:** 10.3390/cells12131720

**Published:** 2023-06-26

**Authors:** Adam Jędrusiak, Wojciech Fortuna, Joanna Majewska, Andrzej Górski, Ewa Jończyk-Matysiak

**Affiliations:** 1 Bacteriophage Laboratory, Hirszfeld Institute of Immunology and Experimental Therapy, Polish Academy of Sciences, 53-114 Wroclaw, Poland; adam.jedrusiak@hirszfeld.pl (A.J.); joanna.majewska@hirszfeld.pl (J.M.); andrzej.gorski@hirszfeld.pl (A.G.); 2 Department of Neurosurgery, Wroclaw Medical University, Borowska 213, 54-427 Wroclaw, Poland; wfortuna@onet.pl; 3 Phage Therapy Unit, Hirszfeld Institute of Immunology and Experimental Therapy, Polish Academy of Sciences, 53-114 Wroclaw, Poland; 4 Infant Jesus Hospital, The Medical University of Warsaw, 02-006 Warsaw, Poland

**Keywords:** antibiotic resistance, blood–brain barrier, central nervous system, cerebrospinal fluid, cognitive processing, gut–brain axis, gut phageome, intranasal delivery, therapeutic phages, phage display, phage therapy

## Abstract

The central nervous system manages all of our activities (e.g., direct thinking and decision-making processes). It receives information from the environment and responds to environmental stimuli. Bacterial viruses (bacteriophages, phages) are the most numerous structures occurring in the biosphere and are also found in the human organism. Therefore, understanding how phages may influence this system is of great importance and is the purpose of this review. We have focused on the effect of natural bacteriophages in the central nervous system, linking them to those present in the gut microbiota, creating the gut-brain axis network, as well as their interdependence. Importantly, based on the current knowledge in the field of phage application (e.g., intranasal) in the treatment of bacterial diseases associated with the brain and nervous system, bacteriophages may have significant therapeutic potential. Moreover, it was indicated that bacteriophages may influence cognitive processing. In addition, phages (via phage display technology) appear promising as a targeted therapeutic tool in the treatment of, among other things, brain cancers. The information collected and reviewed in this work indicates that phages and their impact on the nervous system is a fascinating and, so far, underexplored field. Therefore, the aim of this review is not only to summarize currently available information on the association of phages with the nervous system, but also to stimulate future studies that could pave the way for novel therapeutic approaches potentially useful in treating bacterial and non-bacterial neural diseases.

## 1. Introduction

Numerous papers have suggested that gut bacteria play a critical role in shaping our health [1,2,3]. It has been demonstrated that a healthy gut microbiota is essential for proper development, immune response, homeostasis, and even learning processes [4,5,6,7,8,9]. Besides the bacterial component of the microbiota, other crucial microorganisms are viruses—more specifically, bacteriophages (phages), which are bacterial viruses, estimated to outnumber bacteria in the gut by one order of magnitude (10^15^ phages and 10^14^ bacteria) [10,11,12]. Interestingly, it is probable that 31 billion phage particles pass across the human gut epithelial cell layers each day [13]. The influence of bacteriophages on human health and their role in shaping the overall homeostasis have yet to be fully understood, but it is acknowledged that phages may have a huge impact on the composition of gut microbiota and the function it serves [14,15,16]. 

Bacteriophages are viruses that invade bacteria. Not only can they eradicate these bacteria, but they also play a significant role in bacterial evolution. This is due to the bacteriophages’ ability to integrate their genetic material with the bacterial genome and facilitate the transfer of genes between bacteria. Phages are ubiquitous, found in both the environment and living organisms [17,18], and may be isolated from different sources, ranging from aquatic samples to soil, or from human specimens [19,20]. Interestingly, they occupy nearly every ecological niche within our bodies, predominantly inhabiting areas such as the urinary tract, lungs, gut, mouth, and skin [21,22], and the composition of each body niche phageome varies significantly [21]. Among different human phageomes, those inhabiting the gut and mouth are probably the most thoroughly characterized because these sites are easily accessible and phages occur there in high concentrations [19,23,24,25]. 

The central nervous system (CNS) is composed of the brain (cerebrum and cerebellum), spinal cord, and optic nerves, as well as the membranes covering them, and can be infected by different pathogens, regardless of difficulties in penetrating the blood–brain barrier (BBB) [26]. The system is well shielded by the BBB, the major site of a blood–CNS exchange. In humans, it is a neurovascular unit composed of brain microvascular endothelial cells (BMECs), pericytes, astrocytic endfeet, microglia, and neurons. All these cells are connected to each other by tight junctions or adherens junctions, making it extremely difficult for any molecule to penetrate [27]. Surprisingly, recent studies have shown that bacteriophages may also be present in the central nervous system. This is in line with a previous report of bacteriophages’ ability to penetrate the BBB, which opens up a new research area, expanding their therapeutic potential in treating CNS-related bacterial infections, but also in brain cancers and other brain-related diseases (including the study of phages as carriers transporting different drugs through the BBB) [28,29,30,31,32,33,34,35,36].

In this review, we focused on the occurrence of natural bacteriophages in the nervous system, linking their presence in the intestine with the influence they may have on the nervous system. Additionally, we also focused on the current knowledge of phage application and its potential in the treatment of diseases associated with the brain and nervous system (which have presented serious difficulties for treatment). 

## 2. Phage Distribution in the Human Body

### 2.1. Composition of the Gut Phageome

The intestinal microbiota has been shown to play a major role in physiological processes in host organisms and its dysregulation has been linked to the development of many diseases, such as irritable bowel syndrome (IBS), colorectal cancer, obesity, inflammatory bowel disease (IBD), *Clostridium difficile* infection, or neurological disorders [37,38,39,40,41]. The bacterial community, which is a primary component, plays a crucial role in mammalian gut physiology. It facilitates metabolic functions, protects against pathogens, and modulates the immune response [7,8,42]. The human gut is also a place where fungi and viruses—especially bacteriophages—are observed [19,23,24,25]. Their role is to shape the structure of the bacterial community by specific lysis (disintegration or decomposition of the cell wall structure) of particular bacteria, but also by modulating the activity of the immune system, mediating the anti-inflammatory response, as well as playing a protective role against invasion of pathogens [14,16,43,44].

Bacteriophages follow one of two main life cycles: lytic and lysogenic. In the lytic cycle, the complete destruction of bacterial cells is observed. These bacteriophages (lytic, virulent) use host cell machinery to replicate and assemble new phage virions. On the other hand, the lysogenic cycle is favored by temperate phages, which base their life cycles on the ability to integrate their genetic material with the host genome and switch to the latent stage in the form of so-called prophages. Such phages can also enter the lytic cycle in response to certain conditions, lyse the cell, and release progeny virions into the environment. It is reported that the lysogenic cycle is favored by phages inhabiting human and animal bodies [45,46]. By comparison, bacteriophages in aquatic ecosystems prefer the classic “kill-the-winner” dynamic [46,47]. The reason for this is still unclear, but Knowles et al. hypothesized that the polarity between the ecosystem of the human body and aquatic ecosystems is based on the observation that temperate phages become increasingly important in diverse ecosystems, especially those of high microbial densities [48]. These phages can modulate gene expression in bacteria and alter their phenotype through transposition, induction, and horizontal gene transfer (HGT). Temperate phages may also transfer virulence or antibiotic-resistance genes between bacterial cells. The vast repertoire of genes can shape bacterial diversity and function; thus, bacteriophages contribute to bacterial balance and homeostasis. Shifts in the composition of the virome (mainly phageome) affect human health indirectly because some diseases are suggested to be linked to alterations in the bacterial community [49,50,51,52,53,54,55,56,57]. Moreover, phages, including temperate ones, may also modulate eukaryotic cell functions and human immune response by inducing, among others, phagocytosis, cytokine production, and leukocytes activation, as well as the polarization of macrophages [58]. 

Current research suggests that the composition of the gut phageome is highly individualistic, as it consists mostly of long-term colonizing temperate phages [59], but it has also been shown to change throughout the human lifespan. It adapts to our microbiota and changes by affecting not only bacterial hosts but also the human organism by modulating immune response [59,60,61,62,63]. After birth, phage content and diversity are extremely low. However, this changes during the early stages of life, when the human phageome is highly dynamic. Initially, it is dominated by dsDNA tailed bacteriophages, representing sipho-, podo-, and myovirus morphotypes, previously classified (before major changes to bacteriophage taxonomy, reflecting genomic relationships rather than morphology-based classification) as members of—now abolished—*Siphoviridae*, *Podoviridae*, and *Myoviridae*, respectively. Phages belonging to the *Microviridae* family (ssDNA) were also detected. During infancy, viral and bacterial composition undergo dynamic modifications. Interestingly, the greatest richness and diversity of gut bacteriophages is observed within the first four days of life, and subsequently decreases over time, reaching stability in adulthood [64]. Additionally, it can be disturbed by different factors, such as diet, lifestyle changes, or diseases [65,66,67]. The gastrointestinal tract as well as other human body surfaces are lined with a mucosal layer, utilized by microbes to inhabit and to communicate with the host [68]. Bacteriophages populate mucosal surfaces by binding to mucin glycoproteins through immunoglobulin-like spikes present on their capsid in a process referred to as bacteriophage adherence to mucin (BAM). This phenomenon is believed to most likely play two significant roles: protection from pathogenic bacteria for the human host and providing lysogens with an environment to develop a bacterial symbiotic relationship that benefits the human host [62,65,66].

Reyes et al. (2010) performed a metagenomic analysis of human gut viromes and demonstrated that the fecal virome is highly diverse, while interpersonal viral diversity remains relatively low [46]. Moreover, up to 80% of virome reads generated in that study did not match any known sequences in public databases, which highlights the difficulty in identifying bacteriophage communities and the limitations of these estimates. Interestingly, Dutilh et al. (2014) applied a cross-assembly approach for unknown virome sequence data and identified a ~97 kb circular genome of a novel dsDNA phage named “CrAssphage” [67,68]. It is highly abundant and ubiquitous in all human fecal samples amplified using *Bacteroides intestinalis* [69]. Manrique et al. (2016) proposed the term “healthy gut phageome”, which consists of core and common phages present in healthy individuals that are likely globally distributed [61]. According to the authors, it is generally dominated by temperate tailed phages (formerly grouped in the—now abolished—order *Caudovirales*, currently replaced on the higher taxonomic level with the class *Caudoviricetes* [70,71]) and lytic *Microviridae* bacteriophages [72,73], while in phage dysbiosis the gut phageome has an increased number of lytic phages and/or activated prophages [74]. The change in phageome composition may stimulate the development of life-threatening diseases or progression into a more severe state of diseases, such as periodontal disease, Parkinson’s disease, type 2 diabetes, cancer, or gastrointestinal disease [74,75,76,77,78,79]. Interestingly, it has been observed that there is a significant decrease in core bacteriophages in patients with gastrointestinal diseases such as Crohn’s disease or ulcerative colitis [61]. At the same time, the occurrence of these diseases was correlated with the expansion of pathogenic Proteobacteria (e.g., *Escherichia coli*, *Fusobacterium*) and reduced number of protective bacteria (e.g., *Faecalibacterium prausnitzii*, *Rumininococci*) [80,81,82]. An increasing number of research articles suggest that alterations in the gut phageome may be associated with diet, inflammatory bowel disease, malnutrition, or obesity, but it is important to highlight that it is still unknown whether they are simply a consequence of changes in the gut microbiota or they are directly implicated in these pathological states [63,83,84,85]. 

### 2.2. The Gut Microbiota–Brain Axis Network and Interdependence

There is evidence that gut microbiota can also modulate functions of the nervous system and impact the maintenance of its homeostasis [86,87,88,89,90,91]. The link directly presenting this dependence is the vagus nerve, which maintains physical bidirectional communication between the CNS, intestinal wall, and enteric nervous system (ENS) [92], consisting of neurons and glial cells, distributed throughout the gastrointestinal tract and responsible for controlling coordinated smooth muscle contractile activity and other gut functions [93]. It has been suggested that the development and function of the ENS are to some extent mediated and modulated by gut microbiota [92]. Communication between them involves both direct and indirect pathways. The direct pathway includes altered intestinal permeability and relies on the release of signaling molecules into the gut lumen from immune and enterochromaffin cells. As a result, motor, sensory, and secretory modalities of the gastrointestinal tract increase [94,95,96]. Besides the ENS, those signaling systems consist of the endocrine-immune system, the hypothalamus–pituitary–adrenal (HPA) axis, and both the sympathetic and parasympathetic arms of the autonomic nervous system (ANS) [94,95,97]. Chemical signaling also occurs through a direct and indirect pathway. A well-reported direct pathway includes short-chain fatty acids (SCFAs), which are lipids produced in the fermentation of dietary fiber by microorganisms. They were proven to take part in regulating neuroplasticity, epigenetics, gene expression, and even immune response in CNS [98]. Other examples of chemicals produced by gut microbiota include hormones, such as corticotrophin-releasing hormone (CRH), serotonin, dopamine, and other neuropeptides [5,99,100,101]. It is also worth mentioning that it was demonstrated that increased uptake of fructose or exposure to SCFAs, such as acetic acid, increases the titer of phages specific to *Lactobacillus reuteri* and members of the genus *Lactococcus* [102]. The indirect impact includes modulating the neuroendocrine system [103]. An example of such regulation may include the production of glucagon-like peptide-1 (GLP-1), which was reported to increase appetite. Gut microbiota can also induce the production of neurotransmitters and regulate their concentration, which makes them mediators of classical signaling in the nervous system [104,105,106,107]. Moreover, some intestinal bacteria may also synthesize neurotransmitters—e.g., *Bacteroides*, *Parabacteroides*, and *Escherichia* spp. produce γ-aminobutyric acid (GABA) [104], which is the main inhibitory neurotransmitter in the human cortex [108]. As stated above, the gut phageome contains mostly CrAssphages specific to *Bacteroides*, thus it provides an extra layer of regulation in the production of these neurotransmitters. Research has also confirmed that the gut microbiota may suppress certain gut–brain signaling pathways through microbial metabolites. As for the immune response, the gut microbiota plays a crucial role not only in the proper modulation and functioning of the peripheral immune system but also in the maturation, development, and activation of microglia, which is essential for the innate immune response of brain cells [109,110]. The disruption of the bottom-up (gut-brain) motif can have a direct, substantial effect on the host’s mental state, potentially leading to depression, anxiety, and symptoms of bipolar disorder. It may also play a role in the pathogenesis, behavioral disturbance, and progression of neurodegenerative disorders, such as multiple sclerosis, Alzheimer’s disease, and Parkinson’s disease [19,111,112,113,114], as well as amyotrophic lateral sclerosis (ALS) [115] and epilepsy [116].

On the other hand, the top-down (brain–gut) motif is based on the alteration of the gut by disturbed brain homeostasis. Scientists have pointed out several immunological and metabolic markers that are altered during neuroinflammation and infection [117]. It was suggested that if the gut–brain axis exists, then alterations in the CNS should be reflected in urine. Theoretically, during chronic CNS bacterial infection, the gut microbiome would be altered, thus leading to a perturbed metabolism both in the CNS and the gut, eventually releasing modified levels of metabolites or even new metabolites into the bloodstream. These metabolites would be filtered by the kidneys and, consequently, should be present in the urine. To confirm this hypothesis, the levels of three immunological biomarkers—interferon-gamma (IFNγ), vascular endothelial growth factor (VEGF), and myeloperoxidase (MPO)—were examined as their levels are altered during *Mycobacterium tuberculosis* meningitis [118,119]. The studies demonstrated elevated levels of all three biomarkers, indicating that the alteration in the gut–brain axis is indeed detectable in the urine [120,121,122].

### 2.3. Naturally Occurring Bacteriophages in the Central Nervous System

**Figure 1:**
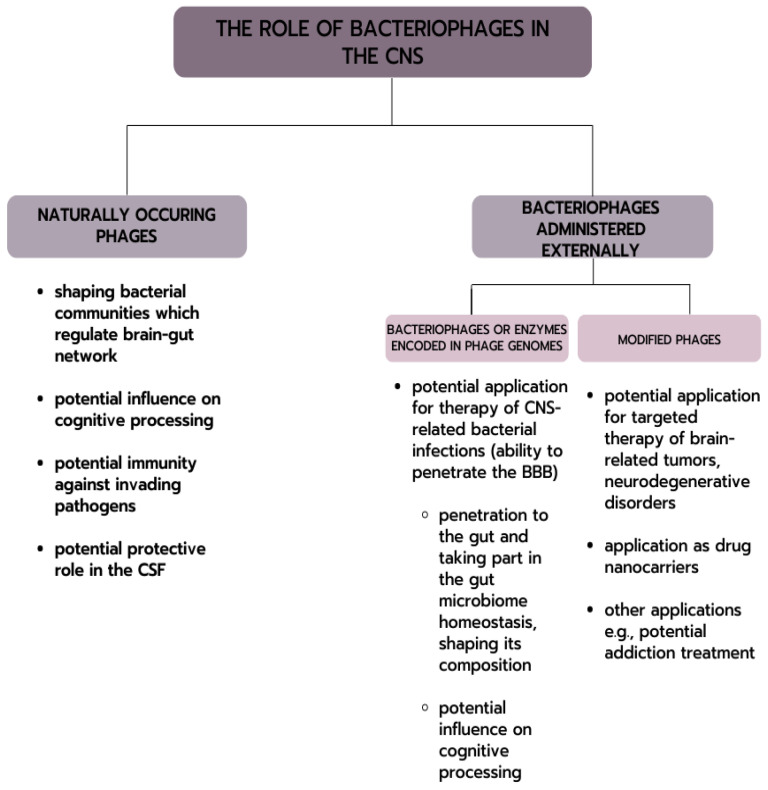
Possible phages interactions with CNS.

Bacteriophages inhabit various sites of the human body. It has been suggested that there is phage translocation from the intestinal tract to other parts of the body [123,124]. It has already been confirmed that they can enter through the epithelial cell barrier by transcytosis [13,125]. This evidence may provide a mechanism by which gut bacteriophages may access previously unknown body sites, such as the nervous system. Interestingly, phages have also been shown to penetrate neural endothelial cells [13]. Figure 1 presents the possible interactions of bacteriophages with the nervous system.

Surprisingly, in 2019, Ghose et al. identified viral particles in the cerebrospinal fluid (CSF) of healthy individuals (which at that time had been commonly considered sterile and unreachable) [28]. Examination of the CSF by epifluorescence microscopy demonstrated virus-like particles (VLPs) at an average of 10^4^ per milliliter, yet other non-sterile human specimens such as saliva contained much more VLPs (10^6^ VLPs/mL). Interestingly, attempts to amplify the V3-4 region of 16S rRNA in order to assess the CSF samples for the presence of any bacterial host were unsuccessful, suggesting that the CSF specimens were bacteria-free. Examination of sequencing reads from the CSF against currently available viral databases revealed that the reads had ~70% homology to known viral sequences, yet further characterization of the putative taxonomic composition determined that most of the contigs (~93%) could not be assigned to any known family, and of those that could be assigned, the vast majority represented tailed bacteriophages. These results suggest that the CSF and probably other parts of the nervous system may be populated by bacteriophages. However, it is still unknown whether these viruses serve a functional role as a non-host-derived immunity against potential bacterial invaders or if they simply entered the CSF with no apparent function [13,28,123,126,127]. Moreover, the effect that phages applied therapeutically may have on the functions of the nervous system has not been described so far.

As phages were confirmed to be naturally present in the nervous system, a new research field emerged to verify whether phages could be used in the treatment of bacterial diseases of the nervous system, such as meningococcal encephalitis, or—as modified bacteriophage—provide a safe carrier to be used in the context of treating cancers such as glioblastoma.

## 3. Bacteriophages and Cognitive Processing

To study the interplay between the gut phageome, the gut bacteriome, and executive function, which is one of the six key domains of cognition, Mayneris-Perxachs et al. performed fecal shotgun metagenomics and metabolomics analyses in four human cohorts [128]. They also tested mice that had received fecal microbiota transplantations and *Drosophila melanogaster* after bacteriophage supplementation. All human participants and animals underwent cognitive tests. As a result of these studies, it was observed that the level of tailed bacteriophages previously classified in the—now abolished—order of *Caudovirales* (now replaced with the class *Caudoviricetes* [70,71]) was negatively correlated with trial-making test B time (TMTB) (a neuropsychological test that involves visual scanning and working memory and examines the executive functions of the brain), while the level of *Microviridae* was positively correlated. In TMTB, participants had to connect numbers and letters in an alternating progressive sequence, which means 1 to A, A to 2, 2 to B, and so on, with precision and time being the main priorities in this test. Higher completion times indicate worse executive functions [129,130]. Sex was also identified as a significant factor when it comes to gut microbiota composition [131]. It was observed that women had lower scores in TMTB, which also correlated with higher *Caudovirales* levels. Surprisingly, no associations were observed in men. Mayneris-Perxachs et al. also performed the backward digit span test, which measures working memory to assess the participant’s ability to hold information in short-term memory and manipulate that information to produce results [132]. In this case, a higher score indicates better executive performance. This time no association was observed in women. The genomic analysis confirmed that most of the *Caudovirales* were uncultured and uncharacterized, and from those that could be identified, mostly phages infecting *Lactococcus* spp., *Enterobacteriaceae*, *Firmicutes* (e.g., *Eubacterium rectale*), or *Bacteroidetes* (*Prevotella copri*) were detected. These specific *Caudovirales* phages belong to the three described families, *Siphoviridae*, *Demerecviridae*, and *Drexlerviridae*, which comprised the former *Siphoviridae* family. As for the new genome-based *Siphoviridae* family, a negative association between this family and TMTB results was found. On the contrary, *Siphoviridae* were associated with better performance in the Stroop test (which measures the ability to inhibit cognitive interference that occurs when the processing of a specific stimulus feature impedes the simultaneous processing of a second stimulus) [133] and both short- and long-term memory [128]. *Microviridae* phages were associated with significant impairment in executive function in both women and men. Bacteriophages from this family were negatively correlated with *Lactobacillus, Streptococcus,* and *Enterococcus*, while they were positively linked to *Bacteroides* and *Prevotella* species. Conversely, the *Caudovirales* were positively associated with lactic acid bacteria. These viruses were supposedly responsible for better executive functions and information processing speed, but also for long- and short-term verbal memory and general cognition, but surprisingly, these correlations were only observed in men [128]. Researchers also found a connection between the specific level of *Caudovirales* and consumption of dietary products and a fat diet containing medium-chain fatty acids, which is in line with findings that supplementation of medium-chain fatty acids improved synaptic plasticity and cognitive function, both in humans and in mice [134,135]. The studies confirmed that a high sugar diet and high uptake of fructose or exposure to SCFAs increases levels of prophages, which can affect bacterial population by two possible mechanisms: firstly, bacteria are killed as a result of prophage induction and entering the lytic cycle, and secondly, the resulting phage progeny can then kill closely related species of bacteria, thus modulating the bacterial community and levels of metabolites they produce [102,136,137].

## 4. Possible Application of Bacteriophages in the Treatment of CNS-Related Diseases

### 4.1. CNS Infections

Bacteria, viruses, fungi, and parasites have different strategies for invading the CNS. Among bacteria infecting the CNS, the most common are *Mycobacterium spp.*, *Brucella spp.*, *Listeria monocytogenes*, *Neisseria meningitidis*, *Streptococcus pneumoniae*, *Haemophilus influenzae*, *Borrelia burgdorferi*, *Staphylococcus aureus*, and *E. coli* [138,139,140]. The spectrum of bacterial infections is wide, ranging from brain abscesses and septicemia to meningoencephalitis, and they can result from hematogenous spread from another site of the patient’s body, contiguous spread from the upper airways, injury, or surgery. Some bacteria, such as *M. tuberculosis*, can enter the CNS via lymphatic nodes [141]. For newborns, the most threatening bacteria are *Streptococcus agalactiae* and *E. coli*, followed by *S. pneumoniae* and *N. meningitidis* in the first weeks of life [142]. As an effect of bacterial infection, a specific cascade of events is initiated. In particular, events in the brain significantly modulate the feedback effect on the gut microbiota [19,143,144]. 

There are three main strategies for neurotropic pathogens to cross the BBB: (i) transcellular migration; (ii) paracellular migration; or (iii) a Trojan horse mechanism [145]. The mechanism of the transcellular entrance is based on the pathogen’s ability to bind to BMECs. The pathogen is then endocytosed, transported within the vacuole through BMECs, and finally released to the brain tissue. In the paracellular mechanism, the pathogen disrupts tight junctions and/or induces the production of pro-inflammatory cytokines, e.g., TNFα, IL-6, or IL-1β. Bacterial infection induces an inflammatory response via glial mediators, which are crucial in the communication between the host’s immune system and the brain, but also between the gut microbiota and the brain [146,147]. In the Trojan horse mechanism, pathogens infect phagocytes and—hidden inside them—cross the BBB and infect the CNS. Several bacteria, including *E. coli*, group B streptococci, *S. pneumoniae*, *N. meningitidis*, *L. monocytogenes*, and *M. tuberculosis*, cross the BBB transcellularly, but *L. monocytogenes* and *M. tuberculosis* can also cross the BBB via the Trojan horse mechanism [148,149]. 

**Figure 2:**
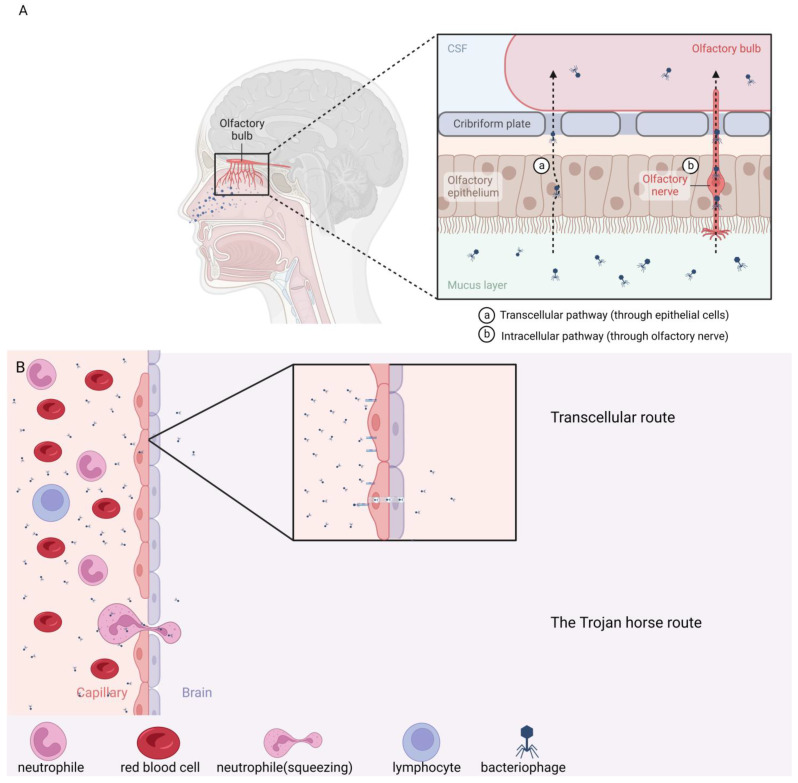
Possible routes of entering the CNS by bacteriophages. (**A**) Intranasal administration; (a) transcellular pathway; (b) intracellular pathway. (**B**) Entering from circulation through the BBB by either transcellular pathway or by the Trojan horse mechanism.

Viruses can infect the CNS either by directly traversing the BBB through one of the three routes described above or by taking nonhematogenous routes. Figure 2 presents possible routes of penetrating the BBB and entering the CNS by bacteriophages. This can be achieved by retrograde axonal transport from peripheral nerves to the CNS, the nasal olfactory epithelium, and neurons. These viruses can stimulate the production of pro-inflammatory cytokines just like bacteria, but they were also reported to stimulate the production of metalloproteases in BMECs, astrocytes, and pericytes. As an effect of the activity of these enzymes, tight junctions can be destabilized by initiating the RhoA kinase pathway and enhancing the permeability of BMEC monolayers in vitro [150,151].

### 4.2. Possibility of Phage Therapy in the Treatment of CNS-Related Bacterial Infections

With dramatically increasing antibiotic resistance, alternative therapeutic strategies are critically needed. Phage therapy (PT) has been considered a vital part of the solution [152,153]. Bacteriophages offer several advantages, including (i) high specificity, allowing selective targeting of pathogenic bacterial strains with no negative impact on the natural microbiota; (ii) self-replication at the side of infection; (iii) the ability to kill antibiotic-resistant bacterial strains; (iv) a lower risk of spreading resistance mechanisms; (v) coevolution with the bacterial host; (vi) safety even for immunocompromised patients; and (vii) little to no side effects [73]. Moreover, the majority of the antibiotics approved over the past decades represent already known classes of antimicrobials, with only a fraction having novel chemical structures [154].

The very first report on using bacteriophages in the treatment of brain-related bacterial diseases dates back to 1943 when Dubos et al. administrated phages specific to *Shigella dysenteriae* intraperitoneally (ip) in a mouse model [155]. What is more, the applied phages were detected in the brains of the animals to which they were administered. After phage application increased, phage titer was observed in infected mice. Interestingly, out of eight mice treated with high bacteriophage titer (10^9^ PFU/animal), six animals survived, whereas out of eight pup rats treated with lower phage titer (10^5^ PFU/animal) only two survived. The next phage breakthrough came in 1982 when Smith and Huggins conducted an experiment using bacteriophages in the treatment of *E. coli* producing colicin V, which causes septicemia and meningitis [156]. They demonstrated that Phage R was highly active in vitro, which was reflected in vivo (when 3 × 10^4^ phage particles given intramuscularly or 3 × 10^3^ given intravenously were sufficient) to cure mice administered a potentially lethal intramuscular dose of *E. coli*. The authors also demonstrated that although phage R-resistant mutants are relatively common in laboratory cultures, they were only found in a few phage-treated mice, and even then they were confined to the inoculation site and outnumbered by phage-sensitive cells. The efficacy of PT vs. antibiotic treatment was compared and showed that one dose of phage R was at least equivalent to multiple doses of streptomycin and more effective than multiple doses of tetracycline, ampicillin, chloramphenicol, or trimethoprim plus sulphafurazole in treating mice infected intramuscularly or intracerebrally with *E. coli*. The therapeutic success of PT was due to high in vivo activity and the failure of phage-resistant mutants to proliferate during treatment [156]. In 1998, Barrow et al. investigated *E. coli* causing septicemia and meningitis-like infection and newly isolated phage R (procured from sewage water) selective for K1 antigen-possessing bacterial strains [157]. This antigen is a capsular polysaccharide, associated with a highly virulent nature, and is often found in pathogenic *E. coli* [158]. When chickens were intramuscularly infected with 10^6^ CFU or intracranially inoculated with 10^3^ CFU of *E. coli* and simultaneously intramuscularly treated with 10^6^ or 10^8^ PFU of phage R (in the case intramuscular or intracranial bacterial administration, respectively), no morbidity or mortality of the animals was observed [157]. The authors administered lower doses of bacteriophage preparation into the chickens and found that in the case of the intramuscular bacterial inoculation, the application of 10^2^ PFU caused some protection against bacteria, but the effect was not statistically significant. However, with the intracranial bacterial administration, doses lower than 10^8^ PFU were not successful. Interestingly, the authors also observed that newly hatched chickens could acquire some protection against pathogens with lower doses of bacteriophages (10^6^ PFU) compared to older chickens (10^8^ PFU). They also tested the effectiveness of PT on colostrum-deprived calves infected orally and demonstrated that in calves treated intramuscularly with phage R, bacterial infection was controlled, yet the infection could not be prevented altogether. Administration of PT delayed the onset of signs of *E. coli* bacteremia.

Poulliot et al. demonstrated that isolated lytic bacteriophages can neutralize *E. coli* strain S242, which causes sepsis and one of the most severe *E. coli* infections, fatal neonatal meningitis, with a mortality rate of up to 25% [30]. The mortality rate may increase as the infection itself may worsen if the pathogen acquires multi-drug resistance (MDR), and *E. coli* strains expressing acquired extended-spectrum beta-lactamases (ESBL), such as CTX-M-type enzymes [159], turned out to be especially lethal. The major one is CTX-M1, which is an enzyme with hydrolytic activity against cefotaxime. The EC200^PP^ bacteriophage (isolated from environmental sewage samples in France) was characterized in vivo and ex vivo with regard to stability and pharmacokinetic properties in pup rats. The EC200^PP^ was stable for at least 24 h in adult and pup rats’ serum, while in human serum phage titer decreased by 2 to 3 logs in the first 2 h of incubation and remained stable after that. As stated in the article, serum possesses phage-inhibitory activity, and this activity is not specific to EC200^PP^, but rather to all phages. When phage preparation was administrated, most of the phage virions were preferably located in the spleen and kidneys, while low titers were observed in urine and the CNS. Because *E. coli* S242 strain may induce sepsis and meningitis, both models were investigated. For the sepsis model, pup rats were injected with 10^4^ CFU of S242 bacteria ip and then administered 10^8^ PFU/mL of EC200^PP^ subcutaneously 7 h or 24 h after the induction of infection. Interestingly, a 7 h post-infection intervention resulted in 100% recovery, while phage administration 24 h after infection resulted in only 50%. For meningitis models, two scenarios were investigated: with a lower dose of the S242 bacteria administered intrathecally, and with a higher inoculum of bacteria, corresponding to that normally encountered in human meningitis. The first case was based on administering 200 CFU per rat to cistera magna, which resulted in a titer of 10^6^ CFU/mL in the CSF after 24 h. Pup rats were then treated with 10^8^ PFU of EC200^PP^ per pup rat administered ip 1h post-infection, which contributed to a 100% survival rate up to day 5. This also contributed to nondetectable bacterial levels in the CSF, and EC200^PP^ titer of 4.5 +/− 0.2 log PFU/mL 24 h post-infection. Based on the highly successful initial results, a second scenario was then investigated, with a higher concentration of S242 bacteria (2 × 10^6^ CFU) administered intrathecally, relevant to that observed in human meningitis, and then 10^8^ PFU of EC200^PP^ 1h post-infection. As a result, all rats survived to day 5 of the experiment. After 24 h, the CSF samples were positive for bacteria, but no signs of bacteremia were observed, and on day 5, in three out of five rats, the CSF was sterile with no signs of infection. However, when phage treatment was delayed to 2 or 3 h post-infection, the survival rate decreased dramatically [30].

Bacteriophage K1F specific to *E. coli* strain EV36 also expressing the K1 capsule antigen was used in an in vitro model of bacterial neonatal meningitis in human cerebral microvascular endothelial cells (hCMEC), which are a part of the BBB [160,161,162,163]. The studies revealed that the K1F phage was internalized by phagocytosis upon contact with hCMEC cells, and then degraded through a constitutive pathway—via lysosomal degradation along with PAMP-LC3-dependent phagocytosis, which suggests that phages are recognized by Toll-Like Receptor 3 (TLR3) on mammalian cells. Importantly, the K1F phage did not induce an inflammatory response. The authors also reported temporarily decreased barrier resistance of hCMEC cells, which could facilitate the transition of immune cells across the endothelial vessel in vitro. Importantly, K1F can infect the EV36 strain intracellularly within hCMEC cells without inducing inflammation [29].

Apart from whole phage virions, bacteriophage endolysins were also recently documented to demonstrate great potential for treating severe cases of infections with gram-negative and gram-positive bacteria, such as *E. coli*, *Salmonella* spp., *Pseudomonas aeruginosa*, *Pseudomonas putida*, *Shigella boydii*, *Shigella flexneri*, *Vibrio fischeri*, *Vibrio vulnificus*, and many more [164,165,166,167,168]. Phage endolysins are subdivided into five families depending on the peptidoglycan disruption mechanism: glucosaminidases, lysozymes or muramidases, lytic transglycosylases, endopeptidases, and amidases. [169,170,171]. Gram-positive bacteria are more prone to endolysins, which are able to disrupt the peptidoglycan layer. Because gram-negative bacteria possess an outer membrane, shielding the peptidoglycan layer, the activity of endolysins towards gram-negative pathogens may be restricted [172] as the low permeability of the outer membrane makes the exogenous application of endolysins challenging [173] and some endolysins require additional strategies to permeate the outer membrane, such as endogenous lysis mediated by holins, pinholins, and bacterial Sec translocases or exogenous lysis [174]. Their medical potential is highlighted by the fact that, unlike for bacteriophages, there are no reports of bacterial resistance to endolysins acquired using classic mechanisms: by mutations, receptor modification, passive adaptation, restriction modification, CRISPR-Cas, and pseudolysogeny [175]. The most suitable and diverse endolysin described to date is Cpl-1 derived from *S. pneumoniae* bacteriophage Cp-1 [176]. Usually, bacteriophages are highly selective for a specific strain of bacteria; however, phage endolysins can display a broader activity against different and multiple hosts [166] and they can be easily engineered to enhance their lytic potential [177]. An increasing number of papers confirm their medical potential [178,179,180,181,182], but the results of clinical trials on phages have yet to prove the formal efficacy of PT.

### 4.3. Phages and Autoimmune Diseases of the Nervous System

Recent data from our and other groups suggest that phages possess immunomodulating activities that may have the potential to control aberrant immune responses, including autoimmune reactions [15,183]. Autoimmune disorders of the nervous system (e.g., inflammatory demyelinating diseases, myasthenia gravis, etc.) pose a significant medical challenge as their treatment is not always satisfactory while side effects of currently available therapy may be serious and sometimes life-threatening [184,185]. Therefore, in those clinical settings one could envisage the potential therapeutic action of selected phages with proven anti-inflammatory and immunosuppressive activity and a virtual lack of side effects [186].

## 5. Potential of Phage Application in Targeted Therapy for Brain Cancers

Glioblastoma—the most abundant and highly aggressive primary brain tumor in adults—is characterized with a rising incidence rate, currently ranging from 0.59 to 5 per 100,000 persons internationally [187], with a median life expectancy of approximately 14–16 months [188], and only 5% of all patients surviving to 5 years post diagnosis [189]. In terms of risk factors, glioblastoma is poorly understood. Due to the low frequency of the tumor worldwide, it was suggested that the brain demonstrates a higher degree of protection from genotoxic stress than other organs [190]. Because brain cells are protected from mutagens by the BBB, it was postulated that brain tumors, especially glioblastoma, arise from a small pool of adult neuronal stem and progenitor cells (NSPC), which are located in the subventricular zone, subcortical white matter, but also in the dentate gyrus of the hippocampus [191,192,193]. These cells retain the ability to enter mitosis and play a crucial role in learning and memory [194]. Of all these cells, glioma-initiating cells or glioma stem-like cells are the most important; they can self-renew, differentiate, and form secondary tumors in xenotransplantation [195]. The progeny of glioma-initiating cells mostly resembles astrocytes, but some cells were also noted to have features of endothelial cells or pericytes [196,197,198]. In most cases of glioblastoma, tumors are located in the frontal, temporal, and parietal lobes, and rarely in the occipital-lobe, cerebellar, brainstem, and spinal cord [189]. The current standard of care involves surgery, followed by radiotherapy in combination with chemotherapy [195]). In recent years, molecular targeted therapy was suggested as an alternative treatment option in glioblastoma, and this topic was comprehensively reviewed by Le Rhun et al. (2019). Possible targets are molecules involved in oncogenic signaling via tyrosine receptor kinases, cell cycle control, and susceptibility to apoptosis. Repurposing of drugs conventionally used in other diseases, e.g., metformin or the anti-epileptic drug valproic acid, is also considered.

Even though many different drugs that potentially target glioblastoma have been investigated, none of them have entered clinical practice, possibly because most glioblastomas are far from a single-pathway driven disease [195]. As reported, glioma stem-like cells are resistant to radiotherapy and chemotherapy for yet undefined reasons [199,200,201,202,203,204,205]. Especially in the case of brain tumors, the therapeutic effect is difficult to achieve because of the presence of the BBB, which impedes the penetration of drug particles. Moreover, the glioma stem cells (GSCs) turned out to be capable of self-renewal and resistant to standard treatment [202]. This provides an unmet need to create innovative solutions that would allow difficulties in the delivery, pharmacokinetics, and therapeutic use of currently available drugs to be overcome, but also the development of new vectors and novel methods of administration, diagnosis, and monitoring of treatment responses. Bacteriophages may have the potential to meet these conditions.

Filamentous, rod-shaped phages such as f1, fd, and most importantly M13 are widely used in a variety of biochemical and biomedical applications. Recent studies suggest that these phages can greatly impact the development of new therapeutic strategies due to their ability to self-assemble into nanoscale structures [203]. Moreover, rod-shaped nanoparticles possess higher selectivity and specificity as well as targeting abilities to endothelial cells and brain endothelium compared to their spherical counterparts [204]. Filamentous capsids are also robust and monodisperse [205,206]. However, the greatest advantage of these phages comes from their genetic flexibility and easy-to-manipulate structure [206]. One recent breakthrough was the use of bacteriophage nanoparticles in neuronal regeneration and the specific development of biomimetic scaffolds for tissue engineering purposes. Therefore, they may have enormous potential in creating bioactive constructs as a scaffold for repairing and regenerating injured tissues in the nervous system.

The phage display method was developed in order to obtain recombinant, exogenous peptides or proteins exposed on the surface of phage virions [207]. This allows for the preparation of a diverse library of phages presenting peptides/proteins that can be screened to select clones with the highest affinity to the target molecule. Nowadays, phage display technology is commonly used for drug development because it allows for an easy and effective screening of large pools of peptides or antibody variable domains. The development of the method paved the way for a wider use of bacteriophages in nanotechnology and biomedical studies [208]. The phage display technique is a promising tool that may be useful in the treatment of glioma. Targeted therapy, cytotoxic effect, and neuroimaging is achievable [209].

Even though there are alternatives and similar platforms, such as yeast display or ribosome display technology, phage display is still considered the best option due to its high diversity and easy handling [210,211,212,213,214].

As stated earlier, the BBB prevents roughly 98% of potential drugs, pathogens, and molecules from entering the CNS. However, bacteriophages modified with the phage display technique, presenting specific receptors on the surface of their capsids, can penetrate the BBB using the Trojan horse mechanism [215,216]. Moreover, the endocytosis-based mechanism was also confirmed for bacteriophages (Figure 2B) [13,123,217]. The *E.coli* bacteriophage PK1A2 was demonstrated to bind to polysialic acid on the surface of human neuroblastoma cells, thus sharing structural similarity with the *E.coli* phage receptor [218,219]. Not only did the phages bind to eucaryotic cells, but they were also internalized into the cells through endocytosis and largely retained infectivity for 24 h [219]. Anand et al. developed a bifunctional viral nanocontainer based on the *Salmonella*-specific bacteriophage P22 [31]. Ziconotide, an analgesic peptide drug derived from a marine snail, was incorporated into the nanocontainer, while a cell-penetrating peptide HIV-tat was displayed on the exterior surface of the phage capsid. Such a modified P22 was successfully transferred into several cell-based models of rat brain microvascular endothelial cells as well as human brain microvascular endothelial cells. Hence, these findings open another alternative route for drug administration [31]. The F88 phage was also reported to easily penetrate the BBB after intranasal administration. One of the most advantageous features of filamentous bacteriophages is their shape and structure. The linear structure of f88 makes it a favorable nanocarrier to penetrate the BBB. After the intranasal administration of f88, phage particles were detected in the olfactory bulb and hippocampus regions of mice brains [220].

A novel approach using a convection-enhanced delivery method (CED) was used by Ksendzovsky et al., in which a high-pressure gradient of bulk flow is generated as a delivery tool for a desired preparation [221]. The tip of an infusion catheter delivers therapeutics directly through the interstitial spaces of the CNS. By applying this, we eliminate the requirement to deliver high, unwieldy therapeutic concentrations, which enables us to achieve therapeutic concentration of the drug in the brain parenchyma [222]. The results from Ksendzovsky et al.’s experiments indicate that CED is a promising method of phage delivery to the brain due to the fact that the M13 bacteriophage was spotted in the white and grey matter of mice brains.

The development of CNS-related diseases is much more challenging than that of non-CNS diseases due to the presence of the BBB, but also the brain–blood tumor barrier (BBTB). Although the structure itself differs from the BBB, both the BBB and BBTB drastically limit the efficient transport of most currently available drugs, mainly due to their molecular size, which surpasses the threshold limiting the molecules that can readily penetrate BBTS and BBB [223,224]. Additional challenges in CNS drug development include the high costs associated with drugs capable of penetrating the BBB and a potentially increased risk of side effects. Compared to other drugs, those targeting the CNS require higher concentrations due to the indirect route to the CNS via the liver and kidneys. These organs tend to remove a significant portion of these molecules from the bloodstream. One approach to improve drug delivery is through receptor-mediated transcytosis, a method that targets drugs to specific receptors expressed in the BBB. To date, two such monoclonal antibody-based constructs—with either a transferrin receptor or insulin receptor—have successfully completed phase II clinical trials [225,226].

During neurological disorders, the expression of different unique receptors that home in on the CNS can be used to improve drug delivery, and such peptides can be selected with phage display technology [227]. To list a few examples, Eriste et al. identified a novel peptide gHo that effectively binds glioma cells [228] and, when covalently conjugated to the cell-penetrating peptide pVEC and doxorubicin, showed promise in the mouse subcutaneous U87 tumor model, although no effect was demonstrated in the intracranial glioma model [228,229,230]. Chen et al. applied phage display to identify an M1 peptide that localized to the brain and was demonstrated to penetrate the BBB/BBTB [231]. Furthermore, conjugation of paclitaxel to the RGD-modified M1 peptide not only allowed for efficient translocation across the BBB, but also greatly improved—typically low—PTX solubility. Importantly, it was the first vector targeting glioma with the ability to travel through the BBB and BBTS. Kim et al. used in vitro and in vivo phage display screens to select glioblastoma targeting peptides and identified a Cadherin 2-binding peptide with the ability to selectively target GSCs over differentiated glioma and non-neoplastic brain cells [232]. Moreover, in a mouse model of intracranially xenografted glioblastoma, the peptide—administered intravenously—specifically targeted intracranial tumors. Phage display technology has also been used to develop nanobodies targeting CA9 [233] and ABCC3 [234], expression of which was detected to correspond with the worse overall survival of patients with glioblastoma. Another approach could involve combining the use of phage display for identification of tumor-targeting peptides and CAR-T therapy for novel modifications of classic CAR-T, which seems to have potential in the development of novel cytotoxic therapies for glioblastoma, as demonstrated by Potez et al. [202].

Importantly, the potential of phage display is not limited to being a useful platform in the drug discovery process—modified phage particles can also be directly applied as targeted delivery platforms, especially because natural phages generally lack native tropism to eukaryotic cells. Przystal et al. report constructing a dual targeting hybrid vector, containing the genome of human adeno-associated virus (AAV) incorporated into the engineered particles of bacteriophage M13 [32]. The vector was intended for intravenous administration, and its dual tumor targeting system relied on, firstly, targeting α_v_β_3_ integrin receptor by phage-expressed ligand RGD4C, and secondly, tumor-specific and temozolomide-induced gene expression from a *Grp78* promotor, acting as suicide gene therapy. The authors demonstrated the potential of this approach in vivo, as the combination of temozolomide and the hybrid vector suppressed the growth of orthotopic glioblastoma in mice.

Many aspects of phage interactions with the nervous system still remain unexplained, i.e., phage binding to the tumor cells, their delivery, and phage titer after its administration (pharmacokinetics). Moreover, it is yet unknown whether they are sufficient to treat tumors in human.

While all these examples focus primarily on the development of novel anti-cancer therapies, it is important to note that the peptides identified with the phage display technology or modified phage particles can also be used for the development of new strategies for the diagnosis and imaging of tumor cells.

## 6. Intranasal Administration

A different approach to the problem of limited penetration through the BBB was suggested by Wan et al., who focused on bypassing the BBB altogether by choosing a different administration route and simultaneously using a peptide-homing tag, which would theoretically allow issues with the migration of drug molecules to the CNS to be overcome [235]. Intranasal application was proposed as a possible efficient alternative, and the M13 phage display platform was used to screen a peptide library for promising carriers that could then be intranasally administered to rats. Clone7 was selected for further examination based on the highest translocation efficiency—it demonstrated an approximately 50-fold higher translocation efficiency compared to the control phage. The 11 amino acids long sequence of the peptide from Clone7 was identified to be as follows: ACTTPHAWLCG. When administered intranasally to Wistar rats, Clone7 was detectable in the brain in a dose-dependent manner after 15 min, reaching maximum after 45 min, and a higher phage titer was detected in the brain than in the liver, spleen, and blood of the examined rats, indicating that Clone7 was able to bypass the BBB and enter the brain directly. Most of the Clone7 phage particles, as well as the peptide itself, were found along the olfactory nerve. Two possible direct pathways may exist to transport administrated substances from olfactory mucosa into the CNS: the olfactory nerve pathway and the olfactory epithelial pathway (Figure 2A) [236]. The results described above indicate that phages displaying the homing peptide entered the CNS through the olfactory nerve pathway [235]. Interestingly, when administered alone and not on a phage particle, the selected peptide—although clearly observed in the olfactory nerve—could not be detected in the brain in the given time frame of up to 8 h. To date, it has only been observed in the case of a single peptide; however, if this was a more general characteristic of nose-to-brain homing peptides, it would further enforce the application of phage particles as nanocarriers for targeted drug delivery to the brain following intranasal administration. Therefore, this discovery opens a possible new route of drug administration in the treatment of bacterial and viral infections of the CNS and also supports the potential of phage-based solutions in the treatment of brain cancers.

There are also studies of phages with two large, different exogenous peptides that were successfully created in a so-called dual phage display system. The first one, alkaline phosphatase, or green fluorescent protein (GFP), was located on the head, and the other, anti-CEA scFv, on the tail of the lambda phage [237]. A similar approach was used by Rajaram et al., who also created a dual-display system [238] in which a biomarker and a short peptide containing the streptavidin-binding motif Histidine-Proline-Glutamine (HPQ) were displayed on the opposite ends of the M13 bacteriophage capsid. This additional second peptide with a high affinity to streptavidin was introduced to allow for sample (phage) purification on streptavidin-conjugated magnetic beads to increase assay sensitivity and decrease background signal. Finally, Fagète et al. reported the selection of bispecific antibodies, which they described as a new frontier in antibody therapy, that also used a dual-phage system, allowing for the co-selection of pairs of antibodies based on the co-engagement of their respective targets [239]. The authors demonstrated that the use of two complementary leucine zipper domains, which later heterodimerize with a high affinity, allowed for the assembly of two antibody fragments—one displayed on the surface of the phage and the other present in the bacterial cell as a soluble component.

Carrera et al. (2004) prepared phage displaying scFv GNC 92H2, which has an affinity to cocaine receptors. Intranasal administration of GNC 92H2-pVIII (1.0 × 10^14^) resulted in detection of the modified phage in the brain of rats and, as expected, the displayed protein has the ability to block the psychoactive effects of cocaine [240].

All these approaches enhance the detection or specificity of targeting and may be useful in creating bacteriophages for biomedical purposes, both diagnostic and therapeutic.

## 7. Conclusions

While PT has recently received greatly increased attention and its prospects appear promising, the potential application of that therapy in treating diseases of the nervous system has rarely been mentioned. Nevertheless, there are some data in the literature and preliminary studies that strongly suggest that the CNS and peripheral nervous system could indeed be targeted by that therapy. Thus, PT could be used as a weapon against bacterial infections of the CNS. Furthermore, recently described immunomodulating and anti-inflammatory effects of phages could pave the way towards their use in treating autoimmune diseases of the nervous system. In addition, phage engineering could allow the use of such modified phages in the fight against some neoplasms of the CNS. It is therefore likely that classical PT as well as “repurposed” PT could provide more efficient means of treatment of both bacterial and non-bacterial diseases of the nervous system.

## 8. Future Perspective

The current state of the research in the treatment of bacterial infections in the CNS indicates that this area is vastly unexplored. While some foundational research has been conducted, there is much potential for broadening the search for phages specific to bacterial strains that commonly infect the CNS. Although partial ground research has been carried out, it will be highly beneficial if a wide search for phages specific to bacteria commonly infecting the CNS is initiated. After that, the next crucial step would be to determine phage pharmacokinetics, host range, stability, and safety. Then, universal and easily monitored models should be created to study penetration through the BBB of natural and modified bacteriophages, as well as phage-based nanocarriers and their effectiveness in delivering drugs to the targeted tissues and cells. One more highly important direction is testing larger groups of subjects with the treatment regimens that show promising results. One future possibility is bacteriophage-based modifications of the composition of gut microbiota to improve the efficiency of cognitive processes Another field altogether is cancer research, where bacteriophages might fit perfectly due to their natural properties and opportunities provided by the phage display technique—yet their potential is still underexploited. The only issue is if the phage constructs would effectively penetrate the BBB and how—if at all—the immune system would be activated by these particles. Excitingly, recent publications in 2022 have linked bacteriophages with CRISPR-like antiviral systems, a groundbreaking development [241,242,243,244,245,246]. Bacteriophage antiviral systems belong to all six known types of CRISPR-Cas systems and occur as divergent and hypercompact antiviral systems. Some of these systems lack key components, which suggests alternative functional roles or host complementation [246]. There are even reports of using the CRISPR-Cas system to genetically modify bacteriophages, thus giving them new mechanisms of action [247], which is an interesting perspective in the context of therapeutic phage applications.

## Data Availability

Not applicable.
